# Association between elevated blood glucose level and non-valvular atrial fibrillation: a report from the Guangzhou heart study

**DOI:** 10.1186/s12872-019-1253-6

**Published:** 2019-11-28

**Authors:** Lu Fu, Hai Deng, Wei-dong Lin, Shang-fei He, Fang-zhou Liu, Yang Liu, Xian-zhang Zhan, Xian-hong Fang, Hong-tao Liao, Wei Wei, Zi-li Liao, Li-hong Tang, Zu-yi Fu, Mu-rui Zheng, Shu-lin Wu, Yu-mei Xue

**Affiliations:** 1 Department of Cardiology, Guangdong Provincial People’s Hospital, Guangdong Academy of Medical Sciences, Guangdong Cardiovascular Institute, Guangzhou, 510080 China; 2 Guangdong Provincial Key Laboratory of Clinical Pharmacology, Research Center of Medical Sciences, Guangdong Academy of Medical Sciences, Guangzhou, 510080 China; 3 Guangzhou Center for Disease Control and Prevention, Guangzhou, 510440 China

**Keywords:** Hyperglycemia, Type 2 diabetes mellitus, Impaired fasting glucose, Non-valvular atrial fibrillation, Sex

## Abstract

**Background:**

To estimate the prevalence of elevated blood glucose level (EBG, including type 2 diabetes mellitus and impaired fasting glucose), and its association with non-valvular atrial fibrillation (NVAF) in Guangzhou, China.

**Methods:**

The population-based follow-up Guangzhou Heart Study collected baseline data from July 2015 to August 2017 among 12,013 permanent residents aged > 35 from 4 Guangzhou districts. Two streets (Dadong and Baiyun) in the Yuexiu District, and one street (Xiaoguwei) and two towns (Xinzao and Nancun) in the Panyu District were chosen as representative of urban and rural areas, respectively. Each participant completed a comprehensive questionnaire, and underwent physical examination, blood sample collection for laboratory testing, electrocardiography, and other evaluations. Multivariable logistic regression analyses were used to estimate the independent association between hyperglycemia and NVAF prevalence.

**Results:**

The prevalence of EBG in overall study population was 29.9%. Compared with residents without EBG, the odds ratio (OR) for AF among residents with EBG was significantly higher (1.94, 95% confidence interval [CI]: 1.40–2.70, *P* <  0.001), even after multivariate adjustment for metabolic abnormalities (OR = 1.60, 95% CI: 1.14–2.25, *P* = 0.007), and driven by women (OR = 1.80, 95% CI: 1.12–2.91, *P* = 0.016).

**Conclusions:**

In Guangzhou, China, prevalence of EBG is high among residents aged > 35 years and associated with a multivariate adjusted increase in prevalence of NVAF overall and in women.

## Background

Atrial fibrillation (AF), one of the most common cardiac arrhythmias in the general population, is associated with increased risk of ischemic stroke/systemic embolism, heart failure, myocardial infarction and mortality from cardiovascular disease [1–3]. Advancing age, type 2 diabetes mellitus (T2D), hypertension, obesity, and myocardial infarction are the major risk factors for the development of AF [4–6]; male, smoking, alcohol intake, dyslipidemia also are risk factors for AF [7].

Abnormal glucose metabolism, characterized by elevated blood glucose level (EBG), is one of the most common metabolic diseases, and mainly includes T2D and pre-diabetes status, i.e., impaired fasting glucose (IFG). Individuals with T2D (summary relative risk = 1.34, 95% CI: 1.07–1.68) [8] or IFG (hazard ratio = 1.16, *P* = 0.017) [9] have a significantly increased risk for new-onset AF.

The prevalence of diabetes in both rural and urban communities of China is increasing rapidly [10]. The extensive overlap between the risk factors for EBG and those for AF, such as obesity, hypertension, and other metabolic diseases [11], may underlie the observed association between EBG and AF [12–14], warranting adequate adjustment for confounders in its evaluation, which is the aim of the present population-based sampling study of the permanent residents in Guangzhou city conducted by The Guangdong Institute of Cardiovascular Disease and approved by the Guangzhou Medical Ethics Committee of the Chinese Medical Association.

## Methods

### Study population

The collection of baseline data in the Guangzhou Heart Study, a population-based follow-up study, began in July 2015 and completed in August 2017 in Guangzhou. Dadong and Baiyun streets from the Yuexiu District were randomly selected to represent the urban areas while Xiaoguwei street, and the towns of Xinzao and Nancun from the Panyu District the rural ones. Permanent residents aged 35 and older from the randomly chosen locations in Guangzhou city were recruited into this study during 3 mobilization rounds of door-to-door visits or telephone contacts and using a randomized multistage cluster sampling. Study details were informed to study participants and written informed consent allowing access to their medical tests and records was provided in advance of enrollment.

Study inclusion criteria were: 1) Guangzhou permanent residents registered in the Guangzhou Household Register; 2) age ≥ 35 years old; and 3) living in the randomly chosen communities for at least 6 months from the day that they enrolled in the survey. Individuals with the following conditions were excluded: 1) mobility difficulties including high paraplegia; 2) mental or cognitive disorders including disturbance of understanding, dementia, and deaf-mutters; 3) pregnant or lactating females; 4) malignant tumors under treatment; 5) floating population including tenants; 6) Guangzhou residents not living in the selected communities for at least 6 months by the day they participated in the survey; 7) non-Guangzhou residents; and 8) non-responders during the 3-round mobilization.

### Data collection and measurement

The trained field researchers, who had received systematic training for questionnaire, physical measurements, and quality control to guarantee standardization of the investigation procedure, conducted a face-to-face interview with the participant who submitted the signed consent described by Deng et al. [15]. A standard questionnaire was used to obtain each participant’s demographic information and relevant data for AF at the baseline survey, including: 1) general demographic and socioeconomic characteristics (including age and sex); 2) personal history of disease (including hypertension, T2D, valvular disease, hyperthyroidism, chronic obstructive pulmonary disease (COPD), hyperuricemia (HUA), and chronic kidney disease (CKD); 3) lifestyle habits (including smoking and alcohol consumption); and 4) methodological details were as reported by Deng et al. [15].

Physical measurements including height, weight, waist circumference and blood pressure (BP) were performed using standard instruments and protocols. Participants took a sitting position for 5 min before the measurement of BP. Used the electronic blood pressure monitor to lay flat on the table, zero, and placed the center of the cuff in line with the heart of the subject. The lower edge of the cuff was 2–3 cm higher than the elbow joint, and the cuff was firmly tied. All the measurements were recorded twice and the mean of the two values was used for analysis.

The fasting blood sampling were arranged in the morning, and the residents were asked to have an empty stomach and a sitting position. Coagulant tubes with blood samples were centrifuged as soon as possible after blood collection at site before transport. Blood samples were stored in a refrigerator at − 20 °C, and transported to a third-party testing institution according to standardized procedures for the testing of relevant blood indicators, such as fasting plasma glucose (FPG), lipid profile, and renal function assessment, among others in 6 h. The FPG was measured by the hexokinase method.

Experienced professionals from The Guangdong Cardiovascular Institute conducted additional evaluations including electrocardiography (ECG) and 24-h single lead ECG monitoring. Each electrocardiogram was performed by well-trained physician and diagnosed by two specifc electrophysiological experts.

### Definitions

AF was diagnosed based on ECG findings (i.e., absence of consistent P waves, presence of rapid, irregular f waves with a frequency of 350–600 beats/min, and an irregular ventricular response) and/or a history of physician-confirmed AF [16, 17]. All residents with AF underwent cardiac ultrasonography to assess for valvular AF. NVAF was diagnosed as per guidelines [18].

T2D was defined as a FPG level ≥ 7.0 mmol/L or a history of physician-confirmed T2D; and IFG was defined as a FPG level of 5.6–6.9 mmol/L in the absence of a previous diagnosis of diabetes per ADA criteria [19]. Participants with T2D or IFG were assigned to the EBG group and the rest to the normal control group.

Metabolic diseases (MetS) was defined as the presence of three or more of the following risk factors: elevated waist circumference (diagnosed as a waist circumference > 90 cm for males and > 80 cm for females); elevated triglycerides (TG) level: > 150 mg/dL (1.7 mmol/L), or drug treatment for elevated TG; reduced high-density lipoprotein (HDL) cholesterol: < 40 mg/dL (1.00 mmol/L) in men and < 50 mg/dL (1.30 mmol/L) in women, or drug treatment for reduced HDL; elevated BP (diagnosed as a systolic blood pressure (SBP) ≥ 130 mmHg and/or diastolic blood pressure (DBP) ≥ 85 mmHg and/or antihypertention drug treatment in a patient with a history of physician-confirmed hypertension); and elevated FPG: FPG > 5.6 mmol/L (100 mg/dL), or previously diagnosed T2D [20]. Hyperuricemia (HUA) was defined as a uric acid level > 420 μmol/L (7 mg/dL) for males and > 360 μmol/L (6 mg/dL) for females. eGFR was calculated by the CKD-EPI formula [21]. Chronic kidney disease (CKD) was defined as eGFR < 60 ml min^− 1^ 1.73^− 1^ m^− 2^.

### Statistical analysis

All statistical analyses were conducted with SPSS 22.0 statistical software (SPSS, Inc., Chicago, IL, USA). Differences between groups were assessed using Student’s T-test for continuous variables and *χ*^2^ test for categorical variables. Univariable logistic regression analysis and multivariable logistic regression analysis were performed to estimate the association between EBG and the prevalence of NVAF. Factorial analysis was used to test interaction effects between age and sex on the prevalence of EBG, and that between sex and EBG on the prevalence of AF. Data are presented as odds ratio (OR) and 95% confidence interval (CI), mean ± standard deviation (SD), or frequency and percentages. All statistical tests were 2-sided, and a *P* <  0.05 was considered statistically significant.

## Results

### Characteristics of the study population

A total of 12,013 residents were enrolled, and 11,634 completed the entire data collection. After excluding 145 individuals with valvular disease, the final study population was comprised of 4047 men and 7441 women with a mean age of 58.2 y. AF was diagnosed in 175 residents: 57 by previous or field ECG, 81 by portable single lead ECG, and 37 by both. The overall prevalence of AF was 1.46%. Among the 175 participants, 31 were diagnosed with valvular disease, therefore, the remaining 144 NVAF were entered into the final data analysis.

The proportion of EBG was 29.9% in the overall population (31.9% in men and 28.9% in women). Individuals with EBG were older, more often men (*P* = 0.001), and more often came from urban, with higher weight, body mass index (BMI), waist circumference, SBP and DBP, and levels of FPG, cholesterol (CHOL), TG, creatinine, low-density lipoprotein cholesterol (LDL), and higher proportions of elevated waist circumference, tea consumption, history of hypertension, HUA, and CKD than the no EBG group (all remaining *P* < 0.001). Participants with EBG also had significantly lower HDL levels (*P* < 0.001). There were no significant differences in height, smoking or alcohol consumption status, or history of hyperthyroidism, or COPD between the two groups (Table 1).

**Table 1:** Characteristics of the Study Population

Characteristics	EBG (*n* = 3440)	No EBG (*n* = 8048)	*P*-value
Men (%)	37.6	34.2	0.001
Age (years)	62.6 ± 10.5	56.4 ± 11.8	< 0.001
Height (cm)	158.3 ± 8.3	158.6 ± 8.2	0.079
Weight (kg)	62.7 ± 11.1	59.5 ± 10.5	< 0.001
BMI (kg cm^− 2^)	25.0 ± 3.6	23.6 ± 3.4	< 0.001
Waist circumference (cm)	87.9 ± 9.9	82.9 ± 9.8	< 0.001
Urban (*n*, %)	1900, 55.2	3503, 43.5	< 0.001
SBP (mmHg)	137.8 ± 20.6	127.4 ± 19.7	< 0.001
DBP (mmHg)	82.7 ± 11.7	79.8 ± 11.5	< 0.001
TG (mmol l^− 1^)	2.1 ± 1.9	1.5 ± 1.2	< 0.001
HDL (mmol l^− 1^)	1.4 ± 0.4	1.6 ± 0.4	< 0.001
CHOL (mmol l^− 1^)	5.6 ± 1.2	5.4 ± 1.1	< 0.001
LDL (mmol l^− 1^)	3.7 ± 1.1	3.6 ± 1.0	< 0.001
FPG (mmol l^− 1^)	7.0 ± 2.2	5.0 ± 0.4	< 0.001
Elevated waist circumference (%)	67.5	48.0	< 0.001
Elevated TG (%)	45.4	28.2	< 0.001
Reduced HDL (%)	12.0	7.1	< 0.001
Elevated BP (%)	76.8	53.1	< 0.001
Non-valvular AF (*n*, %)	65, 1.9	79, 1.0	< 0.001
Hyperthyroidism (*n*, %)	170, 5.0	373, 4.6	0.473
COPD (*n*, %)	203, 6.0	435, 5.4	0.286
Hypertension (*n*, %)	1588, 46.2	1789, 22.2	< 0.001
HUA (*n*, %)	1675, 48.7	2875, 35.7	< 0.001
CKD (*n*, %)	514, 14.9	736, 9.1	< 0.001
Smoking (*n*, %)	763, 22.2	1704, 21.2	0.234
Alcohol consumption (*n*, %)	719, 20.9	1732, 21.5	0.471
Tea consumption (*n*, %)	2607, 75.8	5785, 71.9	< 0.001

Data are presented as mean ± SD or n (%). Elevated waist circumference was diagnosed as a waist circumference > 90 cm for males and > 80 cm for females. Elevated TG was diagnosed as a TG level > 150 mg/dL (1.7 mmol/L), or drug treatment for elevated TG. Reduced HDL was diagnosed as a HDL level < 40 mg/dL (1.00 mmol/L) in men and < 50 mg/dL (1.30 mmol/L) in women, or drug treatment for reduced HDL. Elevated BP was diagnosed as a SBP ≥ 130 mmHg and/or DBP ≥ 85 mmHg and/or antihypertention drug treatment in a patient with a history of physician-confirmed hypertension

*BMI* Body mass index, *CHOL* Cholesterol, *CKD* Chronic Kidney Disease, *COPD* Chronic Obstructive Pulmonary Disease, *DBP* Diastolic Blood Pressure, *EBG* Elevated Blood Glucose, *FPG*, Fasting Plasma Glucose, *HDL* High-density Lipoprotein, *HUA* Hyperuricemia, *LDL* Low-density Lipoprotein, *NVAF* Non-valvular Atrial Fibrillation, *TG* Triglycerides, *SBP*, Systolic Blood Pressure

### Prevalence of EBG among Guangzhou residents

**Fig. 1:**
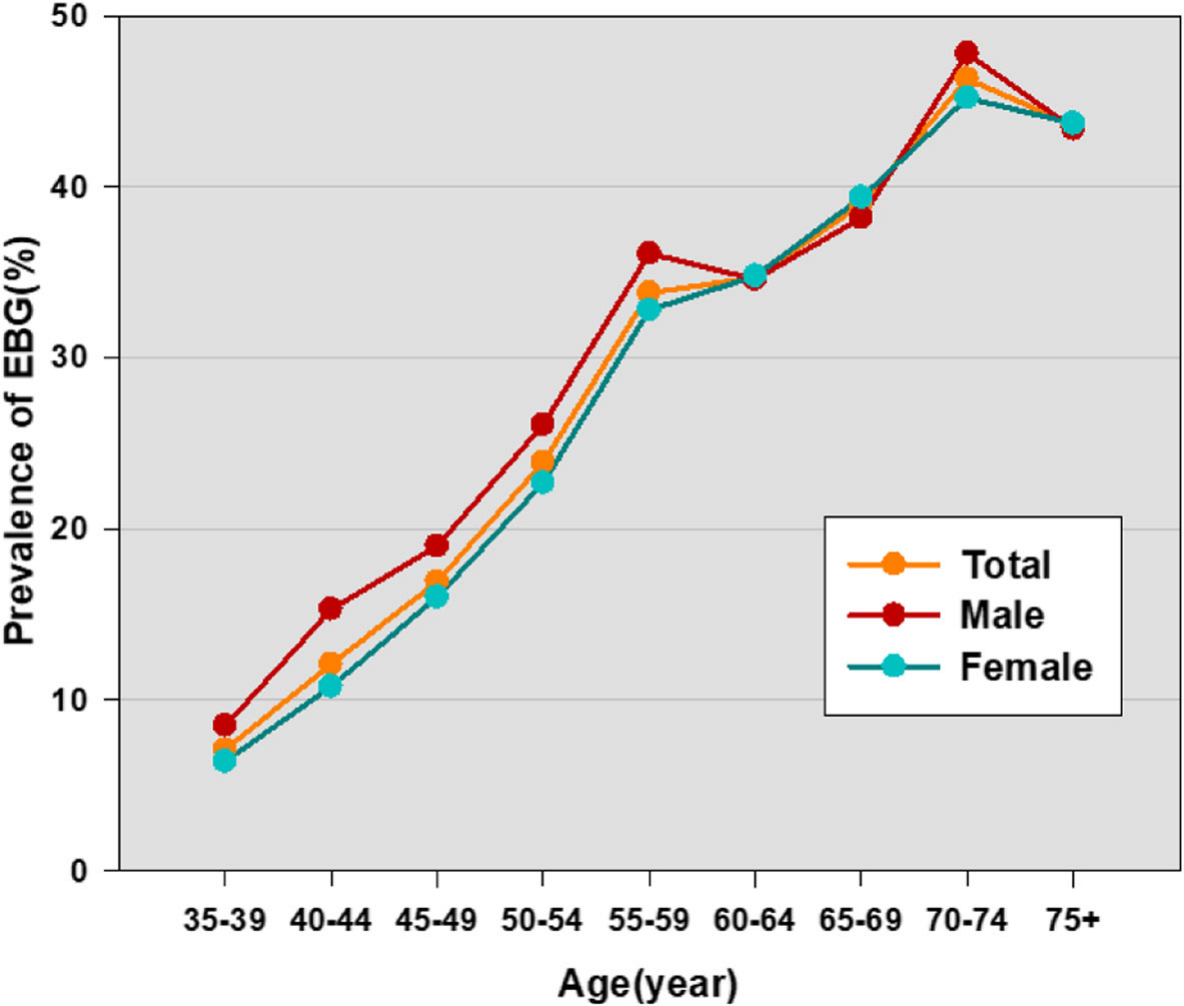
Age-specific prevalence of EBG in the study population. EBG: Elevated Blood Glucose

Prevalence of EBG among Guangzhou residents was 29.9% overall and increased with age in the overall population and similarly in the sex subgroups (Fig. 1). In each age group younger than the 60–64 age group, men had a higher prevalence of EBG than women. The prevalence of EBG was similar for the sex subgroups after age 60–64 age; however, in the 70–74 age range, men had numerically higher EBG than women. There were interaction effects between age-60-year-old and sex in the prevalence of EBG (*F* = 4.183, *P* = 0.041). Using 60 as age cutoff, we included age as a categorical variable into the subsequent statistical analyses.

### Association between EBG and NVAF in the overall study population

As shown in Table 2, individuals with history of hypertension, T2D, hyperthyroidism, or COPD were associated with NVAF significantly. History of T2D had a significantly association with NVAF (OR = 2.33, 95% CI: 1.53–3.56, *P* < 0.001), even after adjusting for age (OR = 1.29, 95% CI: 1.05–1.60, *P* = 0.018).

**Table 2:** Risk Factors for NVAF from Medical History

Risk Factors	Univariable model		Age-adjusted model	
	OR (95% CI)	*P*-value	OR (95% CI)	*P*-value
Hypertension history	3.52 (2.52–4.92)	< 0.001	2.21 (1.56–3.14)	< 0.001
T2D history	2.33 (1.53–3.56)	< 0.001	1.29 (1.05–1.60)	0.018
Hyperthyroidism	2.20 (1.26–3.85)	0.009	2.18 (1.24–3.82)	0.007
COPD	2.80 (1.73–4.52)	< 0.001	2.20 (1.36–3.57)	0.001
Smoking	1.36 (0.94–1.98)	0.100	1.23 (0.85–1.79)	0.273
Alcohol Consumption	0.66 (0.42–1.04)	0.085	0.74 (0.47–1.17)	0.202
Tea Consumption	1.03 (0.71–1.50)	0.919	1.11 (0.77–1.62)	0.575
Urban	1.19 (0.86–1.66)	0.292	1.10 (0.79–1.53)	0.576

*CI* Confidence Interval, *COPD* Chronic Obstructive Pulmonary Disease, *NVAF* Non-valvular Atrial Fibrillation, *OR* Odds ratio, *T2D* Type 2 Diabetes Mellitus

There were a total of 3440 individuals with EBG, including and 1412 with T2D. The association between EBG and NVAF was examined by multivariable logistic regression analysis (Table 3). EBG was significantly associated with NVAF (OR = 1.94, 95% CI: 1.40–2.70, *P* < 0.000; and the age-adjusted OR = 1.44, 95% CI: 1.03–2.02, *P* = 0.032). The association between EBG and prevalence of NVAF remained statistically significant after separately adjusting for waist circumference, BP, HDL, or TG (all *P* < 0.05). In multivariable-adjusted models, EBG remained a significant association with NVAF (OR = 1.60, 95% CI: 1.14–2.25, *P* = 0.007). Thus, EBG was associated with NVAF independently from other metabolic abnormalities in the overall population.

**Table 3:** Risk of NVAF According to the Presence or Absence of Abnormal Glucose Metabolism

Models for the overall population				
Characteristics	Glucose Metabolism			
(*N* = 11,488)	Normal	EBG	T2D	IFG
No. of NVAF/No. of participants	79/8048	65/3440	32/1412	33/2028
Univariable-adjusted model	Referent	1.94 (1.40–2.70) 0.000	2.34 (1.55–3.54) 0.000	1.67 (1.11–2.51) 0.014
Age-adjusted model	Referent	1.44 (1.03–2.02) 0.032	1.65 (1.08–2.51) 0.019	1.29 (0.85–1.95) 0.231
Waist circumference-adjusted model	Referent	1.71 (1.22–2.39) 0.002	2.00 (1.32–3.05) 0.001	1.50 (1.00–2.27) 0.053
BP-adjusted model	Referent	1.65 (1.18–2.31) 0.004	1.94 (1.27–2.96) 0.002	1.44 (0.95–2.18) 0.084
TG-adjusted model	Referent	2.04 (1.46–2.85) 0.000	2.50 (1.64–3.80) 0.000	1.74 (1.15–2.62) 0.009
HDL-adjusted model	Referent	1.92 (1.37–2.67) 0.000	2.29 (1.51–3.48) 0.000	1.66 (1.10–2.49) 0.016
Waist circumference/BP/TG/HDL-adjusted model	Referent	1.60 (1.14–2.25) 0.007	1.87 (1.22–2.87) 0.004	1.41 (0.93–2.14) 0.104
Models for the male population				
Characteristics	Glucose Metabolism			
(*N* = 4047)	Normal	EBG	T2D	IFG
No. of NVAF/No. of participants	41/2754	30/1293	14/873	12/754
Univariable-adjusted model	Referent	1.57 (0.98–2.53) 0.062	2.29 (1.30–4.01) 0.004	0.93 (0.48–1.80) 0.834
Age-adjusted model	Referent	1.26 (0.78–2.04) 0.350	1.87 (1.06–3.30) 0.030	0.84 (0.44–1.62) 0.604
Waist circumference-adjusted model BP-adjusted model	Referent	1.32 (0.82–2.15) 0.256	1.86 (1.05–3.29) 0.034	0.93 (0.49–1.79) 0.834
TG-adjusted model	Referent	1.49 (0.92–2.41) 0.109	2.15 (1.21–3.81) 0.009	1.02 (0.53–1.96) 0.950
HDL-adjusted model	Referent	1.62 (1.00–2.61) 0.048	2.37 (1.35–4.18) 0.003	1.10 (0.57–2.10) 0.779
Waist circumference/BP/TG/	Referent	1.56 (0.97–2.52) 0.066	2.27 (1.29–4.00) 0.004	1.07 (0.56–2.04) 0.842
HDL- adjusted model	Referent	1.33 (0.81–2.17) 0.258	1.88 (1.05–3.35) 0.034	0.93 (0.48–1.80) 0.834
Models for the female population				
Characteristics	Glucose Metabolism			
(*N* = 7441)	Normal	EBG	T2D	IFG
No. of NVAF/No. of participants	38/5294	35/2147	14/873	21/1274
Univariable-adjusted model	Referent	2.29 (1.44–3.64) 0.001	2.25 (1.22–4.18) 0.010	2.32 (1.36–3.96) 0.002
Age-adjusted model	Referent	1.63 (1.02–2.61) 0.041	1.45 (0.78–2.71) 0.245	1.78 (1.03–3.06) 0.038
Waist circumference-adjusted model BP-adjusted model	Referent	1.97 (1.23–3.16) 0.005	1.88 (1.01–3.51) 0.048	2.04 (1.19–3.51) 0.010
TG-adjusted model	Referent	1.78 (1.11–2.85) 0.017	1.69 (0.90–3.16) 0.101	1.85 (1.07–3.18) 0.027
HDL-adjusted model	Referent	2.47 (1.54–3.95) 0.000	2.48 (1.32–4.66) 0.005	2.45 (1.43–4.22) 0.001
Waist circumference/BP/TG/	Referent	2.27 (1.43–3.62) 0.001	2.22 (1.19–4.14) 0.012	2.31 (1.35–3.95) 0.002
HDL- adjusted model	Referent	1.80 (1.12–2.91) 0.016	1.71 (0.90–3.23) 0.099	1.86 (1.08–3.22) 0.026

ORs, 95%CIs, *P*-values were expressed in the model lines of the chart above. *BP* Blood Pressure, *EBG* Elevated Blood Glucose, *HDL* High-density Lipoprotein, *NVAF* Non-valvular Atrial Fibrillation, *TG* Triglycerides, *T2D* Type 2 Diabetes Mellitus

The OR for NVAF among individuals with T2D was 2.34 (95% CI: 1.55–3.54, *P* < 0.001), and that this association was attenuated to 1.65 after adjustment for age. Multivariable adjustment for metabolic factors had little effect on this association estimate (OR: 2.29, 95% CI: 1.51–3.48, *P* < 0.001).

IFG was significantly associated with NVAF (OR = 1.67, 95% CI: 1.11–2.51, *P* = 0.014). Statistical significance of the association was lost after adjustment for age (OR = 1.29, 95% CI: 0.85–1.95, *P* = 0.231) or for other metabolic abnormalities (OR = 1.41, 95% CI = 0.93–2.14, *P* = 0.104).

### Sex difference in the association between EBG and NVAF in the Guangzhou community

The associations between EBG and NVAF in sex subgroups were examined by multivariable logistic regression analysis (Table 3). EBG was not significantly associated with NVAF among men in neither unadjusted model (OR = 1.57, 95% CI: 0.98–2.53, *P* = 0.062) nor multivariable-adjusted models (all *P* > 0.05). Men with T2D had a 2.29-fold greater prevalance of NVAF (95%CI: 1.30–4.01, *P* = 0.004), and the association decreased slightly but remained statistically significant after adjustment for age and other metabolism abnormal factors (all *P* < 0.05). There were no statistically significant differences between men with or without IFG (OR = 0.93, 95% CI: 0.48–1.80, *P* = 0.834), while for women, the OR for those with EBG was 2.29 (95% CI: 1.44–3.64, *P* = 0.001), and for those with T2D was 2.25 (95% CI: 1.22–4.18, *P* = 0.010). However, after adjusting for age and combined metabolic disease separately or as a whole, the significant association for women with EBG remained (overall OR = 1.80, 95% CI: 1.12–2.91, *P* = 0.016), while that for women with T2D did not (overall OR = 1.71, 95% CI: 0.90–3.23, *P* = 0.099). IFG was significantly associated with NVAF among women (OR = 2.32, 95% CI: 1.36–3.96, *P* = 0.002), even after adjustment for age (OR = 1.78, 95% CI: 1.03–3.06, *P* = 0.038) or multivariable adjustment (OR = 1.86, 95% CI: 1.08–3.22, *P* = 0.026). There were no interaction effects between sex and EBG in the prevalence of NVAF (*F* = 0.030, *P* = 0.863).

## Discussion

### Main findings of the study

The present study documented that: 1. EBG (T2D and IFG) was not rare among adults in Guangzhou, China, with 29.9% prevalence (31.9% for men, and 28.9% for women); 2. Presence of EBG was associated with a significantly higher (1.9-fold, and 1.6-fold or 1.4-fold after adjustment for combined metabolic comorbidities or age) prevalence of NVAF than that among individuals with normal glucose metabolism; and 3. EBG was significantly associated with NVAF presence in the overall population, and in women, mainly those with IFG.

### Association between abnormal glucose metabolism and prevalence of NVAF

Diabetes mellitus, a strong, independent risk factor for AF, is one of the most rapidly growing chronic illnesses in the world [1, 2, 22–25]. The twofold increased prevalence of NVAF among individuals with EBG in this study is consistent with the 1.8-fold risk increase reported in previous studies on the southwestern [26] and northern [7] populations of China. Increased association persisted after adjustment for metabolic confounders.

EBG includes T2D and IFG. In a previous meta-analysis, individuals with T2D were at significantly increased risk for new-onset AF (summary relative risk = 1.34, 95% CI: 1.07–1.68) [8]. In our study, T2D was significantly associated with NVAF (OR = 2.34) even after adjustment for other metabolic diseases. IFG, as a pre-diabetes condition, is common worldwide and usually not treated with hypoglycemic agents. IFG has been associated with a significantly increased risk of new-onset AF (hazard ratio = 1.16) [9, 27], and in our study, IFG was significantly associated with the prevalance for abnormal glucose metabolism related NVAF among women.

### Effect of sex on the relationship between EBG and prevalence of NVAF

The present study documented a higher prevalence of NVAF among men, which is consistent with previous studies [6, 7, 15], and in contrast to a previous Chinese cohort study in which men did not have increased risk of AF [26]. Previous studies on the southwestern [26] and northern [7] populations of China demonstrated a significant association between diabetes mellitus and AF, but neither mentioned the effect of sex. In the present study, the independent association of EBG and NVAF as well as the predictive value of EBG for NVAF was not apparent among men, which was consistent with previous findings [28].

Multivariable-adjusted models in the present study suggested that IFG was associated with NVAF among women, although female sex was not a risk factor for cardiovascular disease in contrast to male. Therefore, this study would suggest that patients need to be alert about their glycemic status, particularly at the pre-diabetes stage.

### Limitations

The present study has limitations inherent to its cross-sectional design with a relatively small regional sample size which precluded assessment of statistical significance for some age subgroups. Although we tried by various means to capture all evidence to diagnose AF, including AF history, electrocardiogram on the spot, and single-lead 24 h ECG monitor, the AF prevalence might have been underestimated. Underdiagnosis of abnormal glucose metabolism also might have occurred due to long transport time that might have blood glugose levels been underestimated, and the lack of OGTT and undiagnosed IGT and DM based on postprandial glucose levels. Larger, multiregional, randomized controlled studies with long-term follow-up are warranted to further assess the relationship between abnormal glucose metabolism and the prevalence of new-onset AF.

## Conclusions

In this large report from the Guangzhou Heart Study, adults aged 35 and older in Guangzhou, China with EBG had a significantly higher prevalence of NVAF independently of metabolic comorbidities. The significant association between EBG and NVAF was apparent among women but not men. IFG was associated with prevalence of NVAF among women.

## Data Availability

The data are available from the corresponding author on reasonable request.

## Abbreviations

AF: Atrial fibrillation
AUC: Area under the receiver operating characteristic curve
BP: Blood pressure
CHOL: Cholesterol
CI: Confidence interval
CKD: Chronic kidney disease
COPD: Chronic obstructive pulmonary disease
DBP: Diastolic blood pressure
EBG: Elevated blood glucose level
ECG: Evaluations including electrocardiography
FPG: Fasting plasma glucose
HDL: High-density lipoprotein
HUA: Hyperuricemia
IFG: Impaired fasting glucose
LDL: Low-density lipoprotein cholesterol
NVAF: Non-valvular atrial fibrillation
OR: Odds ratio
ROC: Receiver operating characteristic
SBP: Systolic blood pressure
SD: Standard deviation
T2D: Type 2 diabetes mellitus
